# Controlled Synthesis of Mesoporous Solid Polymer Electrolyte Au(Pt)NiCe/C Membrane Electrode for Electrocatalytic Hydrogenation

**DOI:** 10.3390/mi16040436

**Published:** 2025-04-03

**Authors:** Shaqin Wang, Yunhao Feng, Liangming Duan, Yueming Shang, Huaihang Fan, Ji Liu, Jiahao Han, Xiaoqi Wang, Bin Yang

**Affiliations:** 1 Faculty of Materials Science and Engineering, Kunming University of Science and Technology, Kunming 650093, China; 15085360663@163.com (S.W.); 18215116316@163.com (L.D.); 17693179804@163.com (Y.S.); 18342865631@163.com (H.F.); 18869513590@163.com (J.L.); 15819359371@163.com (J.H.); wxq-man@163.com (X.W.); 2 Qingdao Hengxing University of Science and Technology, Qingdao 266100, China; 17863677359@163.com

**Keywords:** ion-beam sputtering, combined electrochemical dealloying, mesoporous structure, Au-based solid polymer electrolyte membrane electrode, cyclohexene hydrogenation

## Abstract

This study presents a structurally tunable Au-based solid polymer electrolyte (SPE) membrane electrode with significantly enhanced performance in organic hydrogenation reactions. Compared to a Pt-based counterpart, the Au-based electrode achieved a 277% increase in cyclohexane yield and a 4.8% reduction in hydrogen evolution during cyclohexene hydrogenation, demonstrating superior catalytic selectivity and energy efficiency. The improved performance is attributed to synergistic optimization of the electrode’s nanostructure and electronic properties. The Au-based electrode exhibited a 215% increase in specific surface area (SSA) relative to its initial state, along with a markedly enhanced electrochemical active surface area (ECSA). These enhancements stem from its mesoporous architecture, lattice contraction, and high density of zero-dimensional defects. X-ray photoelectron spectroscopy (XPS) revealed a negative shift in Au4f binding energy, a positive shift in Ni^0^ peaks, and an increased concentration of oxygen vacancies (Ov), indicating favorable modulation of the surface electronic structure. This reconstruction promotes H* adsorption and accelerates the hydrogenation reaction, serving as a key mechanism for catalytic enhancement. The core innovation of this work lies in the coordinated engineering of nanoscale structure and surface electronic states, enabling concurrent improvements in reaction rate, selectivity, and energy efficiency. These findings offer valuable guidance for designing noble metal-based membrane electrodes in advanced hydrogen energy conversion and storage systems.

## 1. Introduction

The use of hydrogen energy and other renewable energy sources to replace fossil fuels can help mitigate the energy crisis and environmental pollution challenges. The integration of proton exchange membrane (PEM)-based water electrolysis for hydrogen production with electrocatalytic technologies for converting unsaturated hydrocarbons such as benzaldehyde [1], furfural [2], and aromatic hydrocarbons [3] can achieve a simpler and more environmentally friendly hydrogen production/storage process, compared to traditional methods such as microbial and chemical reduction methods [4,5,6].

Precious metal catalysts such as Au and Pt have been widely used in electrocatalytic hydrogenation due to their excellent catalytic performance. Au is known for its low overpotential and strong ability to suppress the hydrogen evolution side reaction (HER) [7,8,9], making it a promising candidate for selective hydrogenation. However, the catalytic activity of Au is often limited by the relatively low number of active sites. Incorporating Ni introduces a synergistic effect [10,11], which not only modulates the electronic structure of Au to enhance hydrogen adsorption but also promotes the overall hydrogenation reaction. This strategy helps reduce the amount of noble metal required while maintaining high activity.

Similarly, Pt exhibits intrinsically high catalytic activity for hydrogenation reactions. Its surface atomic layers show ligand and strain effects, and alloying with Ni further reduces the hydrogen adsorption free energy and increases the number of exposed active sites [12,13]. Nevertheless, the high cost and limited availability of Pt pose challenges for large-scale applications. To further enhance catalytic performance, the incorporation of cerium (Ce), a variable-valence rare-earth element, introduces abundant surface oxygen vacancies [14]. These vacancies facilitate hydrogen molecule activation, improve electron transport, and contribute to greater catalyst stability and dispersion.

Therefore, the rational design of bimetallic AuNi and PtNi alloys [8], combined with Ce doping, allows for the construction of ternary AuNiCe/C and PtNiCe/C catalysts. This approach synergistically integrates the advantages of each component—noble metal activity, Ni-induced electronic modulation, and Ce-enhanced surface structure and reactivity—resulting in improved hydrogen adsorption, more accessible active sites, and superior electrocatalytic hydrogenation performance.

Current preparation methods for Au- and Pt-based thin-film catalysts include coprecipitation [15], sol–gel [16], and ion beam sputtering [17]. Among these, the ion beam sputtering (IBS) method produces Au- and Pt-based thin film catalysts with a denser film layer. During the electrochemical dealloying process, different anionic acids exhibit distinct effects on the specific surface area (SSA) and electrochemical active surface area (ECSA) of thin-film catalysts [18,19]. Based on the above, this study synthesized AuNiCe/C and PtNiCe/C thin-film catalysts on carbonaceous supports using ion beam sputtering (IBS). To obtain porous structures, the materials were modified via electrochemical dealloying in HCl and HClO_4_ solutions. The catalysts were then thermally bonded with a Nafion membrane to fabricate SPE-AuNiCe/C and SPE-PtNiCe/C membrane electrodes. Physicochemical characterization methods were used to analyze the effect of the catalytic phase and surface composition on the hydrogenation performance of cyclohexene. Additionally, the relationship between the composition and structure of the noble metal catalysts and their catalytic performance was investigated in detail. The combination of Au or Pt with Ni forms alloy phases that modulate the electronic structure through ligand and strain effects, enhancing hydrogen adsorption and activation. The introduction of Ce contributes additional surface oxygen vacancies due to its variable-valence states (Ce^3+^/Ce^4+^), which improves charge transfer and catalyst stability. Furthermore, morphological features such as surface roughness, defect density, and SSA are critical factors influencing the number of active sites. These synergistic effects between composition and structure directly affect the electrocatalytic performance, as discussed in the following sections.

## 2. Experiments

Using the IBS technique along with Pt, Au, Ni, and Ce targets, AuNiCe/C and PtNiCe/C thin-film catalysts were prepared at 210 and 350 °C, respectively (the preparation parameters are shown in Table 1). The carbon carriers, including graphite fiber cloth, pure carbon film on a gold mesh, and polished graphite sheets, were sequentially subjected to ultrasonic cleaning for 10 min in 0.5 M H_2_SO_4_, analytical-grade acetone, and ultrapure water (see Table S5 in the Supporting Document). By independently controlling the concentrations of HCl and HClO_4_, temperature, and dealloying time, orthogonal electrochemical dealloying experiments were conducted on the thin-film catalysts. This resulted in the optimal porous AuNiCe/C (A and B series, as shown in the Supporting Document, Table S1) and PtNiCe/C (C series and D series, as shown in the Supporting Document, Table S3) thin-film catalysts. Using optimized HCl and HClO_4_ conditions, the orthogonal electrochemical dealloying of gold and platinum groups was carried out with HCl followed by HClO_4_, and HClO_4_ followed by HCl. (For the A–B series and C–D series, see Tables S2 and S4 of the Supporting Document.) The optimal conditions for the AuNiCe/C sample were determined to be 0.35 M HCl at 50 °C for 15 s, followed by 0.35 M HClO_4_ at 50 °C for 60 min (A-B-1). For the PtNiCe/C sample, the optimal conditions were 0.2 M HCl at 40 °C for 30 min, followed by 0.6 M HClO_4_ at 30 °C for 80 min (C-D-1).

The AuNiCe/C, PtNiCe/C, A-B-1, and C-D-1 catalyst samples were immersed in a mixture of 5% Nafion solution, 5% PTFE, and deionized water (1:1:1) for 5–10 min. After immersion, the samples were removed, air-dried at room temperature, and then placed in a muffle furnace under a nitrogen atmosphere being heated at 600 °C for 30 s. The Nafion membrane was pretreated by boiling it separately in 5% H_2_O_2_, 0.5 M H_2_SO_4_ solution, and deionized water for 60 min. After the pretreatment, the treated samples and the Nafion membrane were hot-pressed at 175 °C for 180 s to synthesize the SPE-AuNiCe/C, SPE-PtNiCe/C, SPE-A-B-1, and SPE-C-D-1 membrane electrodes. Each membrane electrode was placed between the cathode and anode chambers for the hydrogenation of cyclohexene. The cathode chamber was filled with 80 mL of H_2_SO_4_ solution at 50 °C, while the anode chamber was filled with a 50 °C cyclohexene–dimethyl sulfoxide mixture (2:1). The schematic diagram of the experimental setup is provided in Supporting Document, Figure S1. The 2 V DC power supply was connected to the anode chamber, whereas the cathode chamber was connected to an electrochemical workstation (CHI 440B, Shanghai, China). Cyclic voltammetry (CV) tests were conducted for the SPE-AuNiCe/C and SPE-A-B-1 catalysts at a scan rate of 50 mV/s within the potential range of −2.1 to 1.6 V (Ag/AgCl), to obtain the hydrogenation peak area (S) and overpotential (Ed). Similarly, CV tests were performed for SPE-PtNiCe/C and SPE-C-D-1 at a scan rate of 50 mV/s within the potential range of −1.8 to 1.6 V (Ag/AgCl), providing the corresponding S and Ed values. The exchange current density (j) was obtained through Tafel tests using the equation $∆E=a+blg\left|j\right|$. A cyclohexene hydrogenation experiment of 3500 s was conducted using bulk electrolysis analysis.

The phase composition of the catalyst was characterized using an X-ray diffractometer (XRD-7000, Shimadzu, Tsushima, Japan) with a Cu Kα X-ray source. The chemical composition and electronic states of the catalyst were analyzed using X-ray photoelectron spectroscopy ((XPS, PHI 5000 Versa Probe II X, Physical Electronics, Chanhassen, MN, USA). The Au, Pt, Ni, and Ce elemental contents were determined using inductively coupled plasma-optical emission spectroscopy (ICP-OES, APSP 2460, Micromeritics Instrument Corp., Norcross, GA, USA). The surface morphology and crystal structure of the samples were characterized by high-resolution transmission electron microscopy (HRTEM) and scanning transmission electron microscopy (STEM) using a Tecnai G2 TF 30 instrument (FEI Company, Hillsboro, OR, USA). The pore structure and specific surface area of the samples were determined using a surface area and pore size analyzer (BET, Quadrasorb SI, Quantachrome Instruments, Boynton Beach, FL, USA). The hydrogenation products of cyclohexene were analyzed by gas chromatography (GC-MS5977A-7890B, Agilent Technologies, Santa Clara, CA, USA).

## 3. Results and Discussion

### 3.1. XRD Analysis

**Figure 1:**
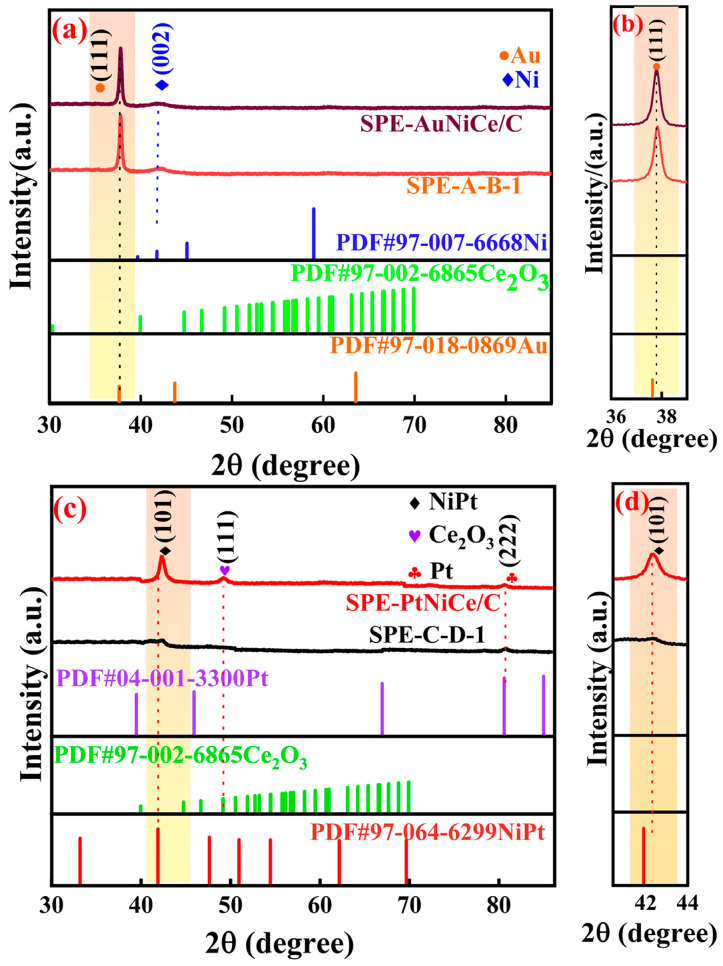
(**a**) XRD patterns of samples SPE-AuNiCe/C and SPE-A-B-1; (**b**) magnified view of the (111) crystal plane of the corresponding samples SPE-AuNiCe/C and SPE-A-B-1; (**c**) XRD patterns of samples SPE-PtNiCe/C and SPE-C-D-1; (**d**) magnified view of the (101) crystal plane of the corresponding samples.

Figure 1a shows the overlaid XRD patterns of the SPE-AuNiCe/C and SPE-A-B-1 samples. In the pattern of SPE-AuNiCe/C, the diffraction peaks at 2θ = 37.6° and 41.78° corresponded to Au (111) and Ni (002) crystal planes, respectively. No AuNi alloy phase was detected, likely due to the formation of an infinite solid solution between Au and Ni [20]. In contrast, the Au (111) diffraction peak of SPE-A-B-1 shifts toward a higher angle, as illustrated in Figure 1b, accompanied by an increase in peak intensity, indicating improved crystallinity. Additionally, no Ce-related diffraction peaks were detected in either sample, suggesting that Ce was either present below the detection limit or existed in an amorphous form.

Figure 1c shows the overlaid XRD patterns of the SPE-PtNiCe/C and SPE-C-D-1 samples. In the pattern of SPE-PtNiCe/C, diffraction peaks corresponding to Pt (222), NiPt (101), and Ce_2_O_3_ (111) crystal planes appeared at 2θ = 80.60°, 41.89°, and 40.17°, respectively. The Ce_2_O_3_ (111) peak showed a broad, shoulder-like shape with a relatively large full width at half-maximum (FWHM), indicating poor crystallinity. Similarly, as shown in Figure 1d, the NiPt (101) peak of SPE-C-D-1 shifts to a higher angle compared to SPE-PtNiCe, further supporting lattice compression. This contraction could increase the d-band electron density of Pt, enhancing the electron conductivity and thereby improving the activity of the SPE-C-D-1 catalyst [21].

### 3.2. STEM and EDX Analyses

**Figure 2:**
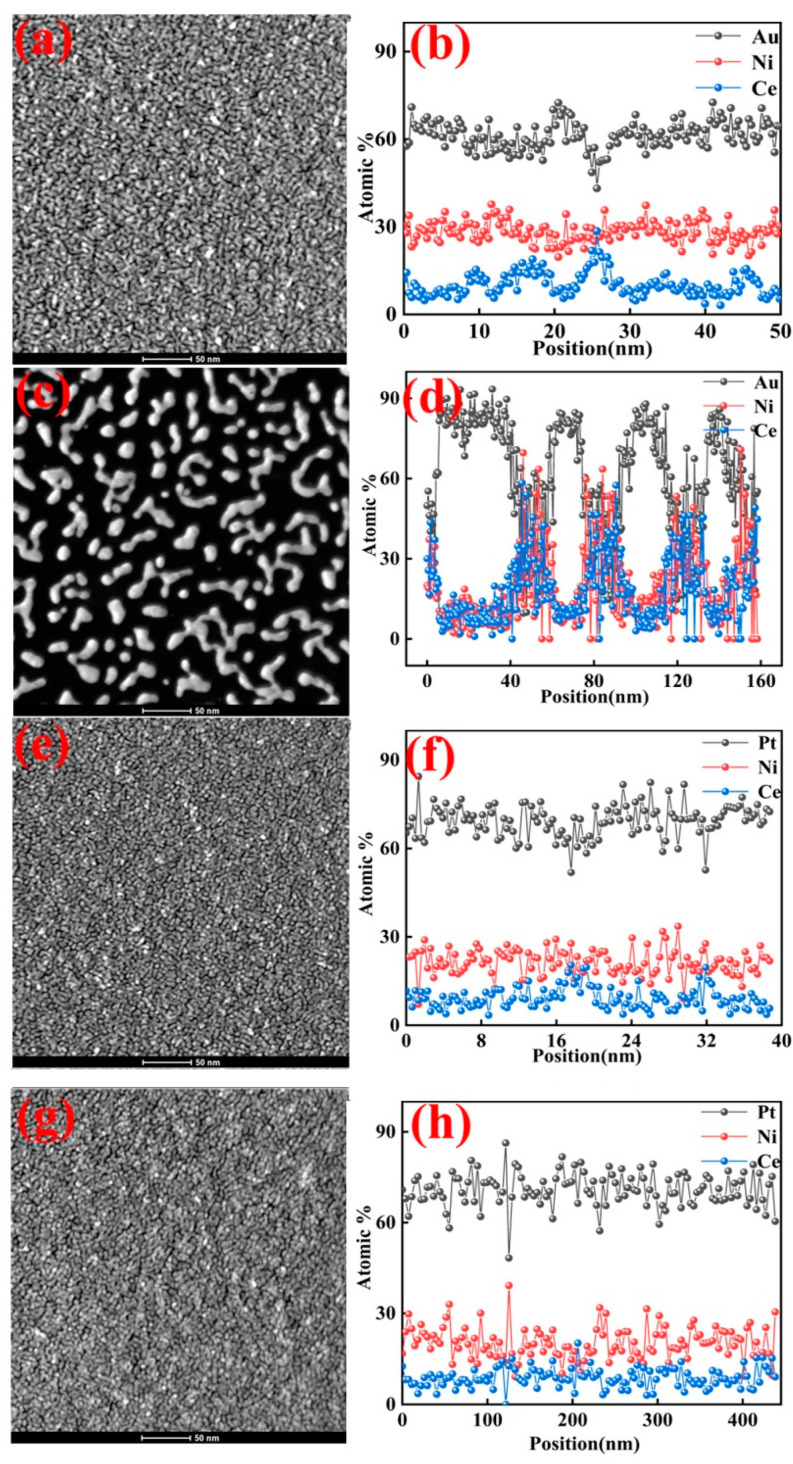
Morphologies and elemental distributions of SPE-AuNiCe/C, SPE-A-B-1, SPE-PtNiCe/C, and SPE-C-D-1 samples; (**a**,**c**,**e**,**g**) STEM images; (**b**,**d**,**f**,**h**) line-scan profiles.

Figure 2 shows STEM images of the above samples. As shown in Figure 2a,b, the surface of the SPE-AuNiCe/C coating had a dense structure, with particles exhibiting spherical, island-like, and dendritic shapes. The three elements showed a uniform distribution, and the Ce doping introduced grain boundaries and a few holes on the surface. As shown in Figure 2c, the entire membrane surface of SPE-A-B-1 exhibited island-like and dendritic structures, mainly composed of Au. The formation of these island structures is the result of the substantial etching of Ni and Ce through the combined electrochemical dealloying process. This surface morphology is expected to significantly increase the surface roughness and SSA value of the membrane electrode. Figure 2d shows that the surface of the particles was primarily composed of Au, with small amounts of Ni and Ce, while the areas between the particles predominantly consisted of Ni and Ce. This distribution significantly enhances the exposed surface area and utilization of Au.

The SPE-PtNiCe/C and SPE-C-D-1 lamina (Figure 2e,g) displayed uniform and compact surfaces, showing only slight morphological differences. Closely packed Pt-rich particles are evident in the figures, while darker, crack-like regions may correspond to grain boundaries formed during atomic deposition. Figure 2f,h show that the Pt atoms on the coating surface exhibited strong corrosion resistance, with only minor etching of Ni and Ce. The surface composition only showed minimal changes. The morphology after the cyclohexene reaction is shown in Figure S2 of the supporting Documents. Since both Au and Pt exhibit excellent stability, the surface morphology remained largely unchanged following electrolysis. Indeed, the post-electrolysis morphology is nearly indistinguishable from that observed prior to electrolysis.

### 3.3. HRTEM Analysis

**Figure 3:**
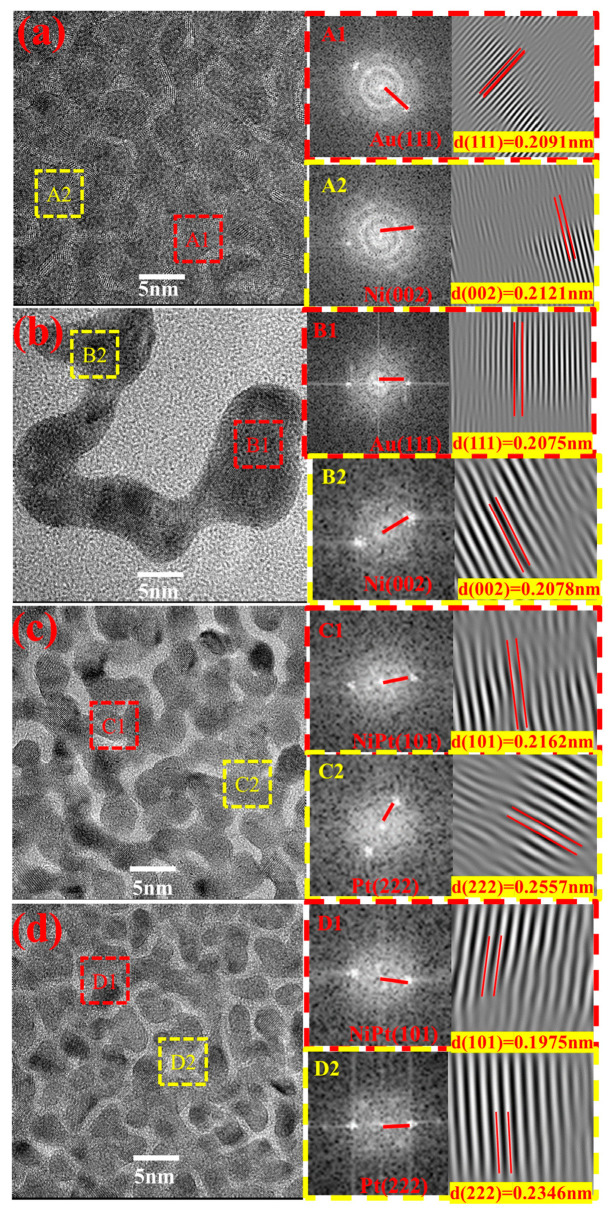
HRTEM analyses of (**a**) SPE-AuNiCe/C, (**b**) SPE-A-B-1, (**c**) SPE-PtNiCe/C, and (**d**) SPE-C-D-1.

Figure 3 presents the HRTEM images of the investigated samples, with crystallographic planes identified for the regions outlined in red in Figure 3a–d. In Figure 3a, the A1 and A2 regions corresponded to the Au (111) and Ni (002) crystal planes, with interplanar spacings (d) of 0.2091 and 0.2121 nm, respectively. The d-spacing of Au (111) fell between the standard values of Au (111) and Ni (002), indicating the formation of an Au–Ni infinite solid solution. The B1 and B2 regions shown in Figure 3b also corresponded to the Au (111) and Ni (002) crystal planes, with d-spacings of 0.2075 and 0.2078 nm, respectively. Both spacings were smaller than the corresponding values of the SPE-AuNiCe/C crystal planes, indicating that the combined electrochemical dealloying process caused Ni and Ce to dissolve from the surface. Most of the branches had hyperbolic structures, and the presence of numerous zero-dimensional defects led to a 0.7% to 2% contraction of the Au lattice [22]. This promoted surface lattice contraction, shortened metal bond lengths, and could enhance the covalent electron density [23,24].

The C1 and C2 regions in Figure 3c correspond to the NiPt (101) and Pt (222) crystal planes, with d-spacings of 0.2162 nm and 0.2557 nm, respectively. Notably, the d-spacing of the NiPt (101) plane (0.2162 nm) lies between those of pure Pt (111) (0.2230 nm) and pure Ni (111) (0.1992 nm). This observation is consistent with Vegard’s law, which states that the lattice parameters of a substitutional solid solution alloy vary linearly with the composition between the lattice constants of the constituent elements. Therefore, the intermediate d-spacing indicates the formation of a substitutional solid solution between Ni and Pt atoms, confirming that alloying has occurred in the NiPt phase. The D1 and D2 regions in Figure 3d also corresponded to the NiPt (101) and Pt (222) crystal planes, with d-spacings reduced to 0.1975 and 0.2346 nm, corresponding to 3.9% to 9.4% decreases, respectively. This also suggests that the combined electrochemical dealloying process promoted the formation of a Pt-rich surface layer with numerous defects [25], leading to lattice compression strain in Pt [26], consistent with the XRD analysis.

### 3.4. XPS Analysis

**Figure 4:**
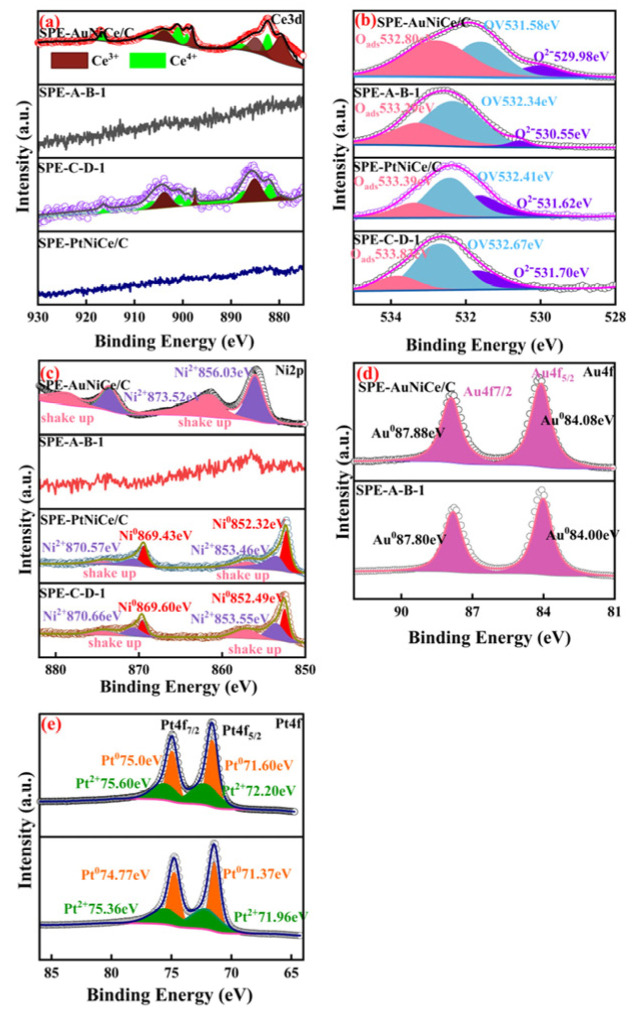
Fitted XPS spectra of SPE-AuNiCe/C, SPE-A-B-1, SPE-PtNiCe/C, and SPE-C-D-1 samples. (**a**) Ce3d; (**b**) O1s; (**c**) Ni2p; (**d**) Au4f; (**e**) Pt4f.

Figure 4a shows the overlaid peak fitting results of the Ce3d spectra of the investigated samples. In the SPE-AuNiCe/C and SPE-PtNiCe/C samples, Ce existed in the form of Ce^3+^ and Ce^4+^ oxides. The Ce^3+^/Ce^4+^ redox pair can easily generate oxygen vacancies within the lattice, which act as electron transport channels in redox reactions, increasing the electron transfer rates and enhancing the catalytic activity [27,28]. Owing to the combined electrochemical dealloying, the Ce content on the SPE-A-B-1 and SPE-C-D-1 surfaces was below the detection limit of the XPS method; hence, no analysis was conducted in these cases.

Figure 4b presents the deconvoluted O 1s XPS spectra of the four samples, including SPE-AuNiCe/C, SPE-PtNiCe/C, SPE-A-B-1, and SPE-C-D-1. In all samples, three distinct peaks can be identified, corresponding to lattice oxygen (O^2−^), oxygen vacancies (OV), and adsorbed oxygen species (Oads). Notably, the peak attributed to O^2−^ located at the lowest binding energy, is primarily associated with Ce–O bonds within the catalyst matrix. After electrochemical dealloying, the lattice oxygen peak intensity in SPE-A-B-1 and SPE-C-D-1 significantly decreased, in line with the substantial reduction in surface Ce content. This suggests that the O^2−^ bonded to Ce also decreased. In contrast, the relative content of OV increased, which is expected to promote improved electrocatalytic activity by enhancing oxygen-related reaction kinetics.

Figure 4c shows the overlaid peak fitting results of the Ni2p spectra of the catalysts. In the SPE-AuNiCe/C sample, Ni was present in the Ni^2+^ oxidation state, with a strong satellite signal near the main peak [29], corresponding to the shake-up peaks of Ni2p [30]. In SPE-A-B-1, the heavy etching of Ni resulted in its content falling below the detection limit of the XPS method; therefore, no analysis was conducted in this case. The Ni2p spectrum of SPE-C-D-1 was split into two peaks corresponding to Ni^0^ and Ni^2+^. Due to the Pt-rich surface of the film, the characteristic Ni2p3/2 peak shifted toward higher binding energies, indicating a transfer of electrons from Ni to Pt [31].

Figure 4d,e present the overlaid peak fitting results of the Au4f and Pt4f spectra of the investigated samples. After the combined electrochemical dealloying, the binding energy of Au4f levels in SPE-A-B-1 showed a negative shift of 0.08 eV compared to that of SPE-AuNiCe/C, whereas the Pt4f binding energy in SPE-C-D-1 displayed a 0.23 eV negative shift relative to that of SPE-PtNiCe/C. This indicates that electrons were transferred from Ni in the alloy to Au and Pt [32,33,34], resulting in an increase in their electron density. This may be due to the dissolution of Ni and Ce from the surface, causing lattice contraction of the primary metals Au and Pt, shortening the Au–Au and Pt–Pt bond lengths, and thus increasing the electron densities of Au and Pt [35].

### 3.5. BET Analysis

**Figure 5:**
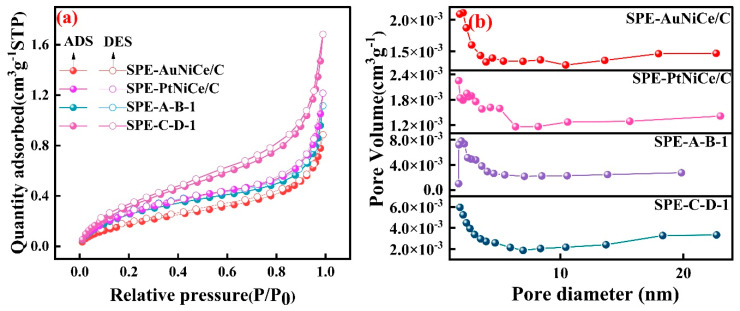
BET analysis of SPE-AuNiCe/C, SPE-A-B-1, SPE-PtNiCe/C, and SPE-C-D-1 samples. (**a**) N_2_ adsorption/desorption isotherms; (**b**) pore size and pore volume curves.

Figure 5a shows the N_2_ adsorption isotherms of the investigated samples, all of which exhibited type IV isotherms with H_4_ hysteresis loops [36]. As shown in conjunction with Figure 5b, the analysis shows that the sample surfaces primarily contained mesopores in the 2–5 nm range, along with a small number of mesopores larger than 5 nm. After the combined electrochemical dealloying, a complex pore structure consisting of numerous micropores and mesopores was formed on the surface of the film electrodes, significantly increasing the pore volume in the 2–5 nm range. The SSA values of the SPE-A-B-1 and SPE-C-D-1 samples increased by 215% and 105% compared to the SPE-AuNiCe/C and SPE-PtNiCe/C (SSA values are shown in Table 2) samples, respectively. Furthermore, the SSA values of Au-based membrane electrodes are significantly superior to those of Pt-based membrane electrodes.

### 3.6. Electrochemical Activity Analysis

**Figure 6:**
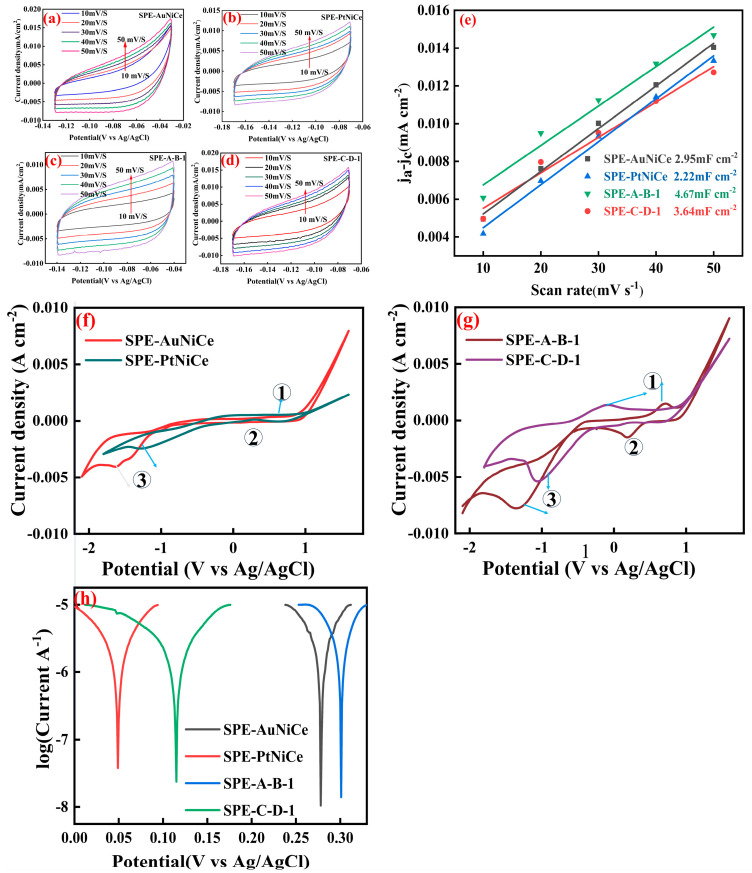
Electrochemical performance of SPE-AuNiCe/C, SPE-PtNiCe/C, SPE-A-B-1, and SPE-C-D-1 samples. (**a**–**d**) CV curves at different scan rates; (**e**) C_dl_; (**f**) CV curves; (**g**) CV curves; (**h**) Tafel curves.

The ECSA can reflect the catalytic activity of the catalyst to some extent as the catalyst’s activity is determined by its electrochemical active surface area [37,38]. By scanning the cyclic voltammograms of the samples SPE-AuNiCe, SPE-PtNiCe, SPE-A-B-1, and SPE-C-D-1 [Figure 6a–d], the linear relationship between the current density difference and the scan rate was established, facilitating the determination of the double-layer capacitance. As shown in Figure 6e, since the electrochemical active surface area is proportional to the double-layer capacitance (C_dl_), the effective electrochemical active surface area of the catalysts can be thus evaluated by the double-layer capacitance. The C_dl_ of the samples SPE-AuNiCe, SPE-PtNiCe, SPE-A-B-1, and SPE-C-D-1 were 2.95 mF cm^−2^, 2.22 mF cm^−2^, 4.67 mF cm^−2^, and 3.64 mF cm^−2^, respectively. This indicates that after electrochemical alloying and dealloying, the electrochemical active surface area of the samples increased, and the electrochemical active surface area of SPE-A-B-1 is larger than that of SPE-C-D-1.

Figure 6f,g display the CV curves related to the hydrogenation reaction. During the cathodic scan (from −1.0 to −1.5 V vs. Ag/AgCl), a distinct hydrogenation peak is observed. The integrated area under this peak (denoted as S) represents the number of active sites participating in the hydrogenation process; therefore, S is positively correlated with hydrogenation activity.

Furthermore, Figure 6f,g illustrate the electrocatalytic behavior of different catalysts in more detail. Specifically,

Peak ① corresponds to the oxygen evolution reaction (OER):

2H_2_O → 4H^+^ + O_2_↑ +4e^−^

Peak ② corresponds to the hydrogen evolution reaction (HER):

2H^+^ + 2e^−^ → H_2_↑

Peak ③ is attributed to the electrochemical reduction of cyclohexene:

C_6_H_10_ + 2H^+^ + 2e^−^ → C_6_H_12_

Compared to SPE-AuNiCe and SPE-PtNiCe, the SPE-A-B-1 and SPE-C-D-1 samples exhibit significantly enhanced peak currents for both oxidation and reduction processes. This improvement is primarily due to the formation of a nanoporous surface structure during the electrochemical dealloying process, which increases the number of active sites and consequently boosts the ECSA. These structural optimizations accelerate the electrocatalytic kinetics, leading to a stronger current response—particularly evident in the cyclohexene reduction reaction corresponding to peak ③.

The integrated area (S) of this peak represents the number of active sites on the catalyst surface [39], indirectly reflecting the effective reaction area of the catalyst. Therefore, S is positively correlated with the hydrogenation activity.

(1) $$∆E=a+blg\left|j\right|$$

In the equation above, ∆E is the overpotential, a is the overpotential at unit current density, b is the Tafel constant, and j_0_ is the exchange current density. Because j_0_ represents the absolute rate of the redox reaction at equilibrium potential, an increase in j_0_ results in a reduction in the required potential and energy consumption to achieve the same reaction rate.

Based on the Tafel curves in Figure 6g, S and j_0_ were calculated using Equation (1); the results are summarized in Figure S3 (in the Supplementary Materials, Figure S3). The S and j_0_ values of the Au-based film electrode were significantly better than those of its Pt-based film electrode. The combined electrochemical dealloying process not only reduced the relative Au content on the surface of the film electrode (as shown in Table 3) but also increased the surface pore volume, suppressing the competing hydrogen evolution reaction [40], thereby enhancing the hydrogenation activity of the catalyst.

### 3.7. Analysis of Cyclohexene Hydrogenation Efficiency via Electrocatalysis

**Figure 7:**
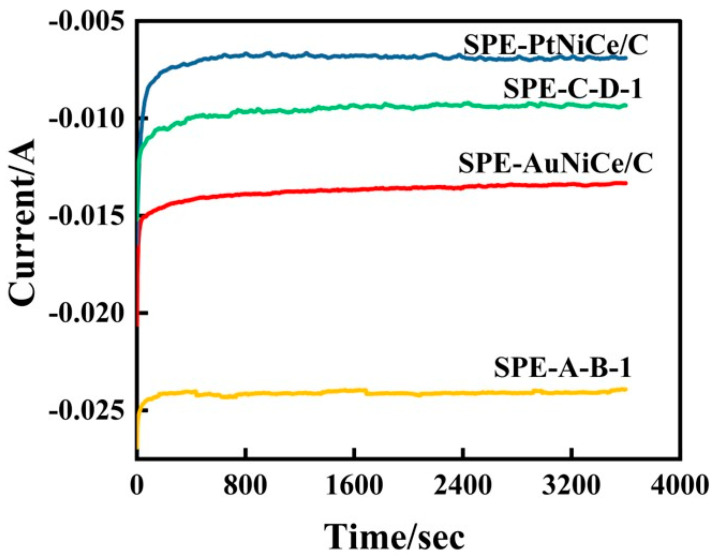
I–t curves for cyclohexene hydrogenation of SPE-AuNiCe/C, SPE-PtNiCe/C, SPE-A-B-1, and SPE-C-D-1 samples.

Figure 7 shows the i–t curve for the hydrogenation of cyclohexene at constant potential by the present samples. The hydrogenation overpotentials for the samples SPE-AuNiCe/C, SPE-PtNiCe/C, SPE-A-B-1, and SPE-C-D-1, as shown in Figure 7, are −1.63 V, −1.79 V, −1.41 V, and −1.05 V, respectively. The corresponding potentials are determined at the hydrogen peak current density maximum, with all electrode areas being 4 cm^2^. The current efficiency of cyclohexene hydrogenation (C) was determined based on the GC-MS analysis and Equation (2), and the results are shown in Table 4.

(2) $$\eta =\frac{Q_{1}}{Q}\times 100\%=\frac{\omega_{C_{6}H_{12}}}{\omega_{C_{6}H_{12}}+\omega_{H_{2}}}$$

In this equation, $\omega_{C_{6}H_{12}}$ represents the production rate of cyclohexane in the hydrogenation reaction, $\omega_{H_{2}}$ denotes the hydrogen production rate in the hydrogen evolution side reaction, Q1 is the hydrogenation current, and Q is the total current.

Figure 7 shows that the current value of the Au-based membrane electrode stabilized at around 50 s, while the Pt-based membrane electrode required approximately 400 s to reach a steady state. Additionally, the i–t curve for the Pt-based electrode exhibited a bend, which may be due to an imbalance between cyclohexene adsorption and cyclohexane desorption on the surface of the electrode, affecting the stability of the hydrogenation reaction.

Moreover, the η values of the Au-based membrane electrodes were superior to those of the Pt-based membrane electrodes. After combined electrochemical dealloying, the $\omega_{C_{6}H_{12}}$ of SPE-A-B-1 increased by 277%, while $\omega_{H_{2}}$ decreased by 4.8%. This indicates that the Au-based catalyst could suppress the hydrogen evolution side reaction. The combined electrochemical dealloying not only enhanced the SSA value of the membrane electrode, facilitating the diffusion of cyclohexene on the surface but also significantly increased the number of active sites on the surface, resulting in excellent cyclohexene hydrogenation conversion and current efficiencies.

## 4. Conclusions

In this work, IBS, combined electrochemical dealloying, and hot-pressing techniques were used to synthesize Au- and Pt-based membrane electrodes for catalytic hydrogenation of cyclohexene. The analysis indicates that the Au-based membrane electrode exhibited a significantly higher catalytic hydrogenation activity for cyclohexene compared to the Pt-based membrane electrode. Both samples contained Au (111), Ni (002), and NiPt (101) phases with interplanar crystal spacings of 0.7% to 3.9%, and Ce was likely dispersed in amorphous form. The surface of the Au-based membrane electrode displayed island-like and hyperbolic branch structures primarily composed of Au, which resulted in a significantly higher surface roughness and increased the SSA value by 215%. A large number of zero-dimensional defects led to Au lattice contraction, resulting in a 0.08 eV negative shift in the Au4f binding energy, while the Ni^0^ peak in the Ni2p3/2 spectrum showed a small positive shift. The η value of the Au-based electrode was better than that of its Pt-based counterpart; moreover, its ESCA value increased, whereas the $\omega_{C_{6}H_{12}}$ and $\omega_{H_{2}}$ parameters increased by 277% and decreased by 4.8%, respectively; these results show that the Au-based electrode has an excellent catalytic hydrogenation activity at low temperatures and effectively suppresses the hydrogen evolution side reaction.

## Figures and Tables

**Table 1:** Ion beam sputtering preparation parameters.

	Parameters	Anode Voltage (V)	Cathode Current (A)	Screen Voltage (kV)	Acceleration Voltage (V)	Beam Current (mA)	Gas Flow Rate (sccm)	Time (s)	Gas Type
Process									
Auxiliary Cleaning		58	14	0.5	200	50	8.8	300	N_2_
Sputtering Preparation		50	12	2.0	140	70	5.2	900	Ar

**Table 2:** Specific surface area values.

Samples	SPE-AuNiCe	SPE-PtNiCe	SPE-A-B-1	SPE-C-D-1
SSA	144.9	174.9	457.7	358.6

**Table 3:** ICP-OES data of SPE-AuNiCe/C, SPE-PtNiCe/C, SPE-A-B-1, and SPE-C-D-1 samples.

Samples	Au (mg·cm^−2^)	Pt (mg·cm^−2^)	Ni (mg·cm^−2^)	Ce (mg·cm^−2^)	Au (%)	Pt (%)
SPE-AuNiCe/C	0.121339	-	0.047167	0.003440	70.60	-
SPE-A-B-1	0.045874	-	0.000408	0.000200	98.69	-
SPE-PtNiCe/C	-	0.128110	0.011250	0.0029901	-	90.00
SPE-C-D-1	-	0.073580	0.001790	0.000270	-	94.24

**Table 4:** Hydrogenation performance of SPE-Pt-M/C and SPE-Au-M/C samples.

Samples	$\omega_{C_{6}H_{12}}$ (%)	$\omega_{H_{2}}$ (%)	η (%)	Q (×10^−3^A)	Q_1_ (×10^−3^A)
SPE-AuNiCe/C	1.53	0.83	64.72	13.55	8.77
SPE-A-B-1	5.77	0.79	88.00	24.09	21.20
SPE-PtNiCe/C	0.85	1.39	38.05	6.86	2.61
SPE-C-D-1	2.34	1.46	61.45	9.40	5.78

## Data Availability

The authors confirm that the data supporting the fundings of this study are available within the article.

## Supplementary Materials

The following supporting information can be downloaded at: https://www.mdpi.com/article/10.3390/mi16040436/s1, Table S1. AuNiCe/C HCl and HClO4 electrochemical corrosion orthogonal. Table S2. Combined electrochemical corrosion of AuNiCe/C HCl and HClO_4_. Table S3. The electrochemical corrosion of PtNiCe/C HCl and HClO_4_ is orthogonal. Table S4. Electrochemical corrosion of PtNiCe/C composite in combined HCl and HClO_4_. Table S5 Experimental materials and Experimental reagents. Figure S1. (a) Cyclohexene hydrogenation apparatus; (b) Schematic diagram of SPE membrane electrode. Figure S2. STEM images of SPE-AuNiCe/C, SPE-PtNiCe/C, SPE-A-B-1, and SPE-C-D-1 after cyclohexene electrolysis. Figure S3. S and j_0_ values.
