# Supplementary material for: Controlled Synthesis of Mesoporous Solid Polymer Electrolyte Au(Pt)NiCe/C Membrane Electrode for Electrocatalytic Hydrogenation
Source: Micromachines (Basel). 2025 Apr 3;16(4):436. doi: 10.3390/mi16040436 (PMC12029946; doi:10.3390/mi16040436)
Supplement: Supplementary file 1 [file micromachines-16-00436-s001.zip › micromachines-3482889-supplementary.pdf]

Supplementary Information

Controlled Synthesis of Mesoporous Solid Polymer Electrolyte Au(Pt)NiCe/C Membrane Electrode for Electrocatalytic Hydrogenation

Shaqin Wang [1,†], Yunhao Feng [2,†], Liangming Duan [1], Yueming Shang [1], Huaihang Fan [1], Ji Liu [1], Jiahao Han [1], Xiaoqi Wang [1] and Bin Yang [1,*]

[1] Faculty of Materials Science and Engineering, Kunming University of Science and Technology,
Kunming 650093, China; 15085360663@163.com (S.W.); 17863677359@163.com (Y F) 18215116316@163.com (L.D.); 17693179804@163.com (Y.S.); 18342865631@163.com (H.F.); 18869513590@163.com (J.L.); 15819359371@163.com (J.H.); wxq-man@163.com (X.W.)

[2] Qingdao Hengxing University of Science and Technology, Qingdao 266100, China; 17863677359@163.com

* Correspondence: yangbin@kust.edu.cn

† These authors contributed equally to this work.

In this paper, the preferred sample electrochemical dealloying conditions are obtained by comparing the magnitude of the values of the hydrogenation peak area (s) and the magnitude of the exchange current density (j_0) (as shown in the table below) under different electrochemical dealloying conditions.

Table S1 AuNiCe/C HCl and $HClO_4$ electrochemical corrosion orthogonal

factor / level	Corrosion systems	Corrosion concentration	Corrosion temperature (℃)	Corrosion time (s)	Hydrogenation peak area (S)	Exchange current density（j_0）
AuNiCe/C					0.559076	0.001259
A-1		0.25	30	20	0.990516	0.001811
A-2		0.25	40	30	1.076101	0.001940
A-3		0.25	50	40	0.931984	0.001826
A-4		0.35	40	20	0.707932	0.001501
**A -5**	**HCl**	**0.35**	**50**	**30**	**1.273190**	**0.002392**
A -6		0.35	30	40	0.850140	0.001759
A -7		0.45	50	20	1.195701	0.002216
A -8		0.45	30	30	1.170104	0.002108
A -9		0.45	40	40	1.118012	0.002154
B-1		0.25	30	2400	0.592924	0.001466
B -2		0.25	40	3600	0.789799	0.002226
B -3		0.25	50	4800	0.571418	0.001433
B -4		0.35	40	2400	0.710132	0.001722
**B -5**	**$HClO_4$**	**0.35**	**50**	**3600**	**1.229301**	**0.002568**
B -6		0.35	30	4800	1.0974565	0.002210
B -7		0.45	50	2400	0.993020	0.002423
B -8		0.45	30	3600	0.705264	0.001571
B -9		0.45	40	4800	0.569715	0.001320

Table S2 Combined electrochemical corrosion of AuNiCe/C HCl and $HClO_4$

sample	combinatorial approach	Combined electrochemical dealloying conditions	Hydrogenation peak area (S)	Exchange current density （j_0）
**A-B -1**		**0.35M-50℃-15s+0.35M-50℃-3600s**	**1.807851**	**0.003429**
A-B-2		0.35M-50℃-15s+0.35M-50℃-1800s	0.608222	0.001851
A-B -3	HCl-$HClO_4$	0.35M-50℃-30s+0.35M-50℃-1800s	1.474006	0.002547
A-B-4		0.35M-50℃-30s+0.35M-50℃-3600s	0.975442	0.001776
B-A-1		0.35M-50℃-1800s+0.35M-50℃-15s	1.390777	0.001944
B-A -2		0.35M-50℃-1800s+0.35M-50℃-30s	1.568064	0.002823
B-A -3	$HClO_4$- HCl	0.35M-50℃-3600s+0.35M-50℃-15s	1.627146	0.002720
B-A -4		0.35M-50℃-3600s+0.35M-50℃-30s	1.440656	0.002480

Table S3 The electrochemical corrosion of PtNiCe/C HCl and HClO4 is orthogonal

factor / level	Corrosion systems	Corrosion concentration	Corrosion temperature (℃)	Corrosion time (min)	Hydrogenation peak area（S）	Exchange current density（j_0）
PtNiCe/C					0.286334	0.000848
C-1		0.2	30	40	0.577916	0.001485
**C -2**		**0.2**	**40**	**60**	**0.829363**	**0.001719**
C -3		0.2	50	80	0.619373	0.001010
C -4		0.4	40	40	0.543159	0.001457
C -5	HCl	0.4	50	60	0.460004	0.001630
C -6		0.4	30	80	0.551390	0.001552
C -7		0.6	50	40	0.757449	0.001595
C -8		0.6	30	60	0.752198	0.001657
C -9		0.6	40	80	0.615836	0.001584
D -1		0.4	30	40	0.266267	0.001567
D -2		0.4	40	60	0.176849	0.001303
D -3		0.4	50	80	0.446938	0.001194
D -4		0.6	40	40	0.536590	0.001337
D -5	$HClO_4$	0.6	50	60	0.372637	0.001265
**D -6**		**0.6**	**30**	**80**	**0.841196**	**0.001877**
D -7		0.8	50	40	0.710975	0.001181
D -8		0.8	30	60	0.558453	0.001262
D -9		0.8	40	80	0.740656	0.001317

Table S4 Electrochemical corrosion of PtNiCe/C composite in combined HCl and $HClO_4$

sample	combinatorial approach	Combined electrochemical dealloying conditions	Hydrogenation peak area (S)	Exchange current density （j_0）
**C-D -1**		**0.2M-50℃-30min+0.6M-30℃-80min**	**1.141030**	**0.002380**
C-D-2		0.2M-50℃-30min+0.6M-30℃-40min	0.976395	0.002086
C-D -3	HCl-$HClO_4$	0.2M-50℃-60min+0.6M-30℃-40min	0.631504	0.001417
C-D -4		0.2M-50℃-60min+0.6M-30℃-80min	0.895279	0.001868
D-C -1		0.6M-30℃-40min+0.2M-50℃-30min	0.911309	0.002005
D-C -2		0.6M-30℃-40min+0.2M-50℃-60min	1.048237	0.002121
D-C -3	$HClO_4$-HCl	0.6M-30℃-80min+0.2M-50℃-30min	0.976825	0.002102
D-C -4		0.6M-30℃-80min+0.2M-50℃-60min	0.812952	0.001624

Table S5 Experimental materials and Experimental reagents

material name	application	Purity	Agency
graphite fiber cloth	multipurpose substrate	>95%	Shanghai Xinxing Carbon Co., Ltd.
carbon-supported gold grid	Substrate for STEM HRTEM	-	EMCN
Au target	target material	≥99.9%	PURI Materials
Ni target/Ni baffle	target material	≥99.9%	PURI Materials
Ce target/Ce baffle	target material	≥99.9%	PURI Materials
high-purity Ar	gas for sputtering ion source	≥99.9％	Messer
high-purity N_2	gas for electrolytic cell	≥99.9％	Messer
anhydrous ethanol	cleaning samples	AR	aladdin
acetone	cleaning samples	AR	Chuandong Chemical
ultrapure water	cleaning samples	-	laboratory preparation
HCl	electrolytic acid etching	AR	Chuandong Chemical
$HClO_4$	electrolytic acid etching	AR	Chuandong Chemical
H_2SO_4	testing	AR	Chuandong Chemical
Nafion solution	curing	AR	DuPont
KCl	for salt bridge reference electrode	AR	Chuandong Chemical
H_2O_2(30%)	cleaning Nafion membrane	AR	Chuandong Chemical
Nafion membrane	SPE support membrane	-	DuPont
cyclohexene	Hydrogenation reactant	AR	Macklin
Dimethyl sulfoxide	hydrogenation solvent	AR	aladdin

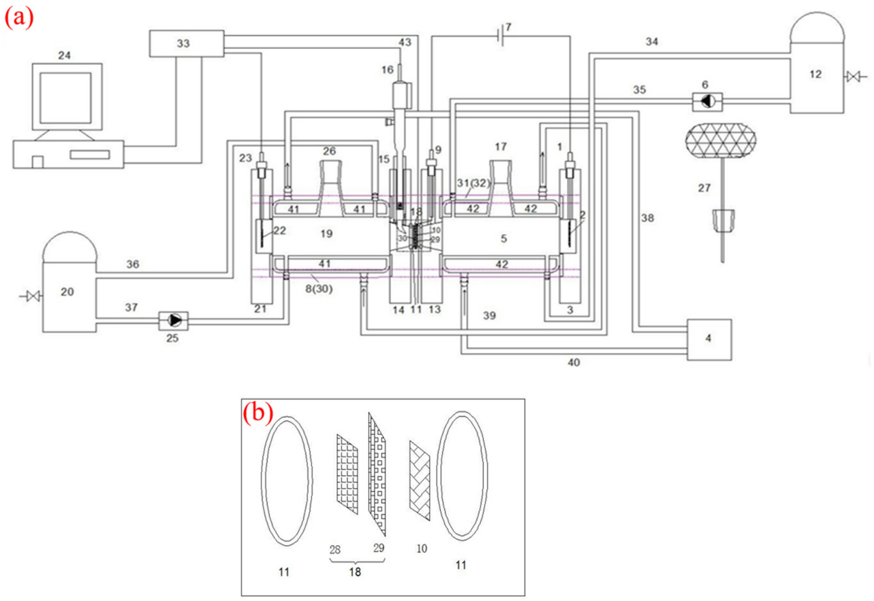

FigS1:(a) Cyclohexene hydrogenation apparatus; (b) Schematic diagram of SPE membrane electrode

1, 9, 23 - Electrode leads, 2, 22 - mesh electrode, 3, 13, 14, 21 - PVC clamps, 4 - Constant temperature water bath circulator, 5 - Anode reaction chamber, 6, 25 - Circulation pump, 7 - DC power supply, 8, 30, 31, 32 - Bolt rods, 11 - O-ring, 12, 20 - Liquid storage tank, 15 - Salt bridge, 16 - Ag/AgCl reference electrode, 17, 26 - Gas collection port, 18 - SPE membrane electrode, 19 - Cathode reaction chamber, 24 - Computer, 27 - Gas collection device, 28 - Catalytic layer, 29 - Nafion membrane, 33 - Electrochemical workstation, 34~40 - PTFE tubing, 41, 42 - Constant temperature water bath, 43 - SPE membrane electrode leads.

STEM images of the samples SPE-AuNiCe/C, SPE-PtNiCe/C, SPE-A-B-1, and SPE-C-D-1 after cyclohexene electrolysis

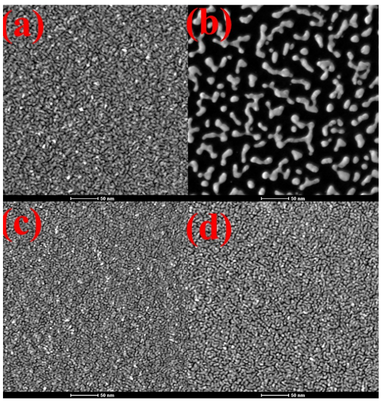

Fig. S2. STEM images of SPE-AuNiCe/C, SPE-PtNiCe/C, SPE-A-B-1, and SPE-C-D-1 after cyclohexene electrolysis.

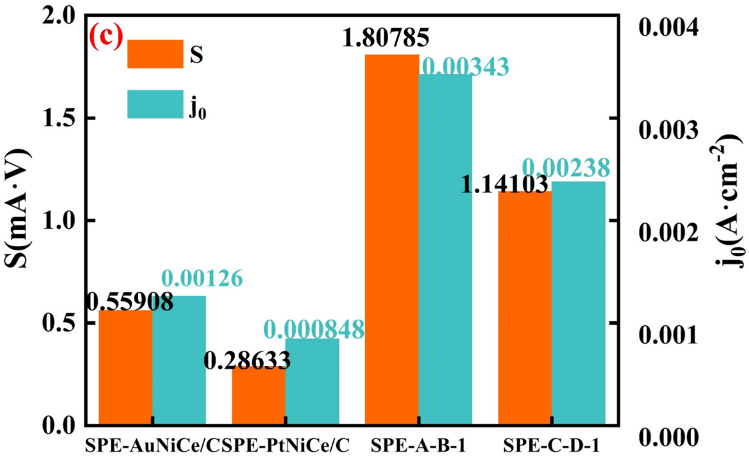

FigS3： S and j_0 values
